# Approaches to Muslim Biomedical Ethics: A Classification and Critique

**DOI:** 10.1007/s11673-023-10239-6

**Published:** 2023-04-19

**Authors:** Hossein Dabbagh, S. Yaser Mirdamadi, Rafiq R. Ajani

**Affiliations:** 1 Department for Continuing Education, University of Oxford, Oxford, OX1 2JA UK; 2 The Institute of Ismaili Studies, Aga Khan Centre, London, N1C 4DN UK

**Keywords:** Muslim biomedical ethics, Contemporary approaches, Classification, Textual, Contextual, Para-textual

## Abstract

This paper provides a perspective on where contemporary Muslim responses to biomedical-ethical issues stand to date. There are several ways in which Muslim responses to biomedical ethics can and have been studied in academia. The responses are commonly divided along denominational lines or under the schools of jurisprudence. All such efforts classify the responses along the lines of communities of interpretation rather than the methods of interpretation. This research is interested in the latter. Thus, our criterion for classification is the underlying methodology behind the responses. The proposed classification divides Muslim biomedical-ethical reasoning into three methodological categories: 1) textual, 2) contextual, and 3) para-textual.

## Introduction

This paper reviews and assesses current methodological approaches to Muslim biomedical ethics. By asking how Muslim scholars tackle biomedical issues in our time at the level of methodology, we aim to identify various methodological approaches among Muslims and to provide a classification and a critique of these approaches. To this end, the classification is principally based on the method of reasoning. Our concern is not with *what* responses Muslim jurists or ethicists arrive at, rather it is with *how* they arrive at those responses, what processes the jurists or ethicists typically undertake to make a legal or a moral judgement, and what approaches they take in their legal or moral reasoning. For example, in addressing a Muslim jurisprudential case on abortion, we are not as such concerned with whether or not abortion is deemed permissible by a jurist. Rather, we are concerned with how a jurist arrives at the ruling (be it permissibility or otherwise), using legal epistemology and hermeneutical toolkits. The proposed classification is thus over and above divisions across denominational lines (Sunnī/Shīʿī) schools of law (*al-madhāhib al-fiqhīyya*), or intellectual traditions (theology, philosophy, and jurisprudence).

Methodology mainly refers to the way in which biomedical-ethical reasoning draws upon the scriptural sources (the Qur’an, the Sunna of the Prophet, and the sayings of the Shīʿī imams). The classification of methodological approaches in Muslim biomedical ethics suggested in this article is based on two criteria: 1) attitude to scriptural text and 2) attitude to rationality. In this sense, the method of classification is mainly about hermeneutical and moral epistemology in contemporary Muslim biomedical ethics.

On the one hand, there are responses that are mainly confined to the “textual” boundaries of the scriptural sources. In textualist methodology, strict compliance with the scripture plays an essential role in reaching judgements about biomedical issues. In its attitude toward rationality, the textualist approach is non-rational, if not necessarily anti-rational. By rational we do not mean the general human capacity for reflection. Nor do we mean by rational what may be understood as coloured by the age of Enlightenment in Europe. Whereas Enlightenment rationality issues a parallel and opposing authority to scripture or religion, rationality as used in this paper does not divest religion of its authority. Here we use rational to mean an independent source for a normative deliberative process, what in Muslim intellectual tradition has been referred to as intellect (*ʿaql*). In Muslim theological circles, especially among the Mu‘tazilites, there has been a tendency to specify the use of speculative reasoning as an intellectual obligation to understand and interpret scriptural sources (*al-wujūb al-‘aqlī li al-naẓar*) (Hanafi 1988, 322ff). Textualists are thus non-rational, not because of their inability to use the human capacity of reasoning but in their decision not to consider human reason as an independent source of deliberation where a scriptural or textual precedent is present. Some textualists either deny reason as a source of knowledge or considerably downplay its epistemic status.

On the other hand, there are responses which are based on a “contextual” understanding of the scriptural sources. In a contextualist methodology, strict compliance with the scripture is also vital, yet a context-sensitive interpretation of the scriptural text is pursued in order to reach judgements about biomedical issues. The contextualist approach is more open toward rationality as a source of knowledge or at least toward rationality as a hermeneutically inevitable tool. While contextualists aim at complying with the text, they give prominence to the study of context of the text in reaching decisions on contemporary biomedical ethical issues. Inasmuch as contextualizing the text requires rationality, contextualists are also rationalist, even though they do not give significant weight to independent reasoning. Reasoning for contextualists remains within the confines of the scriptural text.

Beyond the textualist and contextualist tendencies, a third methodology is emerging among Muslims that can be labelled as “para-textual.” This methodology neither confines itself to the textual boundaries of the scripture, nor does it neglect the scriptural text. What matters most in para-textualist methodology is that contextual interpretations of the scripture are made within the limits of reason. Para-textualists believe in the substantive role of reason, despite knowing that reason is prone to error. Among Muslims, para-textualists are not detached from, or alien to, the ethical vision of the scripture. They do not aim to comply with the text in the way textualists or even contextualists aspire to. However, the fact that they do not neglect the ethical vision of the text is significant enough to make their position relevant to the scripture.

It must be noted that the classification of methodological approaches in this paper is applicable primarily to texts rather than authors. This means that it is both logically and empirically possible for an author to have produced multiple writings, some of which may be classified according to the criteria in this paper as contextualist and some as para-textualist. Thus, it is natural to find some scholars who may oscillate between these approaches in different situations.

## Approaches in Muslim Biomedical Ethics

### Textualist Approaches

#### Analysis

This section elaborates on the common features of a textualist approach to Muslim biomedical ethics. It endeavors to highlight the variety of contemporary textualist approaches among Muslims. The study of biomedical ethical cases from Sunni and Shī‘ī jurisprudence are, by and large, taken to be representative of what can be called the legal textualist approach in contemporary Muslim biomedical ethics.

Textualist approaches are not confined to the legal tradition. One might also think of theological or mystical literalism. However, our concern here is legal/jurisprudential textualist approaches among Muslims. The characteristic feature common to all textualist approaches is that the scripture (the Qur’an and Sunna of the Prophet and that of the Imams) is enough for discerning all we need to know about what morally and legally ought to be done or not to be done. Thus, there is no need for independent substantive reasoning in this regard. Epistemologically speaking, except for logic and linguistics, no considerations independent of the text are needed for understanding the legal rulings of the sacred text. This position can be labelled as epistemological exceptionalism, which means that nothing except the scripture is important for discerning the norms and rules of conduct. From this common ground, the textualist approaches divide along two major lines. For some, the “apparent meaning” (*ẓāhir*) of the sacred text is solely the key for discerning the “intended meaning” (*murād al-mutakallim*) of the scripture (maximal textualism).^1^ Whereas, for others, the apparent meaning is important but not necessarily the only key for understanding the scripture’s intended meaning (minimal textualism). Nevertheless, for textualists of both kinds, the intended meaning of a text is inherent within its apparent meaning, independent of the historical context, the speaker’s intention, or its comprehension by the audience (Gleave 2012).

It is worthwhile to note, that the word *ẓāhir* as used above is a technical hermeneutical term used in the genre of Muslim legal theory (*uṣūl al-fiqh*), which is relevant to, but not the same as, its use in the genre of exegesis (*tafsīr*). *Ẓāhir* in the exegetical genre refers to the exoteric, as opposed to esoteric (*bāṭin*), meaning. In Muslim legal theory, *ẓāhir* (apparent and intended) refers to a multivalent term or statement whose apparent meaning is most probably intended rather than other possible range of non-apparent meanings. There is a constellation of relevant hermeneutical terms used in Muslim legal theory. *Muʾawwal* (non-apparent but intended) is used as an antonym of *ẓāhir* and refers to the meaning of a text that is non-apparent, while being what is most probably intended within the possible range of meanings. *Naṣṣ* (univocal) refers to a text that has an apparent meaning without a range of other non-apparent or other possibly intended meanings. *Mujmal* (equivocal) refers to a text that has no apparent meaning, though having a possibility of multiple intended meanings but without the possibility of preferring one meaning over others. Textualist approaches prefer to work within the hermeneutical categories of *naṣṣ* and *ẓāhir*, over against the categories of *muʾawwal* and *mujmal* to veer towards identifying clear and seemingly unambiguous elements in the scriptural text.

If the apparent meaning of the text turns out to be unreasonable, a maximal textualist will refuse to abandon it in favour of a non-apparent meaning of the text (such as a figurative, allegorical, metaphorical, or contextual meaning). For a maximal textualist, the apparent meaning is to be given priority under all circumstances (Gleave 2012, 146), as it is the only means of discerning the intended meaning of the text. In the history of Muslim legal theory, maximal textualism is associated with a non-extant but still important legal school called “Ẓāhirīyya” (Osman 2014, 3; Sabra 2007, 16). The most prominent Zahiri jurist and theologian was Ibn Ḥazm (994–1064). Ibn Ḥazm believed that the meanings of the Qur’an can be divided into three kinds. Parts that are clear to none, parts that are clear to all, and parts that are clear to some. Parts that are clear to none have no value in deducing legal rulings. The apparent meaning of the rest of the Qur’an is either clear to all or at least clear to some at each time and hence has a legal value. In the Twelver Shīʿī tradition, such an approach is found among the adherents of the legal movement called the Akhbāriyya, which was against the conjecture, opinion, and hadith criticism of their opponent movement Uṣūliyya.^2^ Uṣūlis, at least in theory, gave weight to the opinion of jurists. Even though among contemporary Muslim responses one can hardly find a maximal textualist approach, it deserves to be mentioned so as to understand the widely prevalent minimal textualist approach. The minimal textualists share the conviction with maximal textualists that *in principle* the intended meaning of a text lies in its apparent meaning. This is reflected in what Muslim legal theorists (Uṣūliyya) called “the principality of apparent meaning” (*aṣālat al-ẓuhūr*) (al-Ṣadr 2003, 65). Whereas, for a maximal textualist, this principle is absolute and cannot be compromised, a minimal textualist could, at least in theory, go beyond the apparent meaning under certain circumstances.

A few relevant cases will be presented to show the ways in which a minimal textualist approach can be clearly identified in the works of contemporary jurists who deliberate on biomedical ethical issues. While the selection below is only indicative, a similar textualist approach can be found among most Muslim jurists.^3^ In *Al-Masā’il al-Ṭibbiyya* (Medical Legal Issues), Muḥammad Qāʾinī^4^ takes an approach in dealing with biomedical ethical issues centered on the “principle of permissibility” (*aṣālat al-ibāḥa*), which can be formulated as follows: everything is permissible unless proven otherwise (Qāʾinī 2003). Religious texts in some cases restrict the scope of this principle’s applicability in such a way that if there is no religious stipulation restricting its scope, this principle would remain applicable, otherwise it would be restricted.

To illustrate, Qāʾinī takes the position that after the “coalescence of the semen” (*ʿulūq*), all other things being equal, abortion is impermissible even before the moment of ensoulment (*wulūj al-rūḥ*). Qāʾinī thinks that the principle of permissibility of abortion before ensoulment is overridden by the impermissibility apparent within the scriptural text. Even though he agrees that abortion before the ensoulment cannot be taken as homicide (probably because he thinks that before the ensoulment there is no person, even a potential one), he argues that there are textual precedents (*al-naṣṣ*) forbidding the termination of a fetus from the time of the coalescence of the semen. Thus, the principle of permissibility is to be overridden, and abortion even before ensoulment should be considered impermissible (Qāʾinī 2003). The minimal textualist approach is evident here. Even though there is no person before ensoulment, abortion before ensoulment is to be forbidden on religious grounds, because the apparent meaning of the religious text (according to Qāʾinī) may also be operative as intended in preventing abortion in the early stages of pregnancy.

A similar approach can be found in Ahmad Sahnoun (Ahmad Sahnoun [d. 2003] was an Algerian religious scholar), who argues that the Qur’an and the Prophetic Sunna have placed such a high emphasis on marriage for the protection of individual morals and the multiplication of the community (protection and multiplication) that abortion cannot be considered as religiously permissible (Sahnoun 1974). He makes a claim that anything which leads to the disruption of marriage and to the interruption of procreation is against the purposes which Islam seeks to achieve through marriage. Sahnoun then argues that abortion, among other things, is an instance of interrupting procreation. He is also of the view that the principle of protection and multiplication applies even before ensoulment, making abortion categorically impermissible. Again, the procedure of legal reasoning is in principle similar to the one pursued by Qāʾinī. Both appeal to principles that may or may not be qualified religiously. The only difference is that, while Qāʾinī mentions formal legal principles, such as the “principle of permissibility,” Sahnoun only refers to a substantive scriptural principle, that of protection and multiplication.

Similar minimal textualist approaches can be seen in other areas of biomedical ethics. Muḥammad Āṣif Muḥsinī (Muḥammad Āṣif Muḥsinī [d. 2019] was a Twelver Shīʿī *marja‘* [source of emulation] from Afghanistan), in his work “Islamic Law and Medical Issues,” takes organ transplantation to be categorically impermissible if leading to the death of the donor, immediate or otherwise (Muḥsinī 1984). To substantiate his position, he appeals to a part of the Qur’anic verse that reads “cast not yourselves by your own hands into destruction” (Q2:195)(A. J. Arberry’s translation [Arberry 1996]). He argues that organ transplantation leading to the death of the donor, even if not done willingly and deliberately, would be an instance of casting oneself by one’s own hands into destruction, which he takes the Qur’an to categorically forbid. However, Muḥsinī even considers as forbidden donating a part of the body which may not lead to the death of the donor, identifying it also as an instance of casting oneself by one’s own hands into destruction. Here Muḥsinī states that, although an explicit legal ruling cannot be found to answer this question, it is to be forbidden on the grounds of “Legislator’s preference” (*mazāq al-shāriʿ*). This means that according to Muḥsinī, the scripture displays the apparent preference of the Legislator (God and/or Prophet and Imams) against donating parts of the human body.

The cases above show that in the absence of an explicit legal permission or prohibition, the strategy of the minimal textualist is to extend the textual boundaries rather than to go beyond them, by relying on intended meanings which are apparent within the text. Any appeal to the context or any rational principle outside of the text would not be acceptable to the minimal textualist.

#### Critique

A critique that can be raised against literalism altogether, whether minimal or maximal, is that even minimal (let alone maximal) literalism is based on what is called “semantic atomism” while an alternative theory called “semantic holism” seems more defensible. Semantic holism is anti-literalistic altogether. Semantic holism, or meaning holism, is the view that treats “the meanings of all of the words in a language as interdependent” (Jackman 2020), in such a way that the meaning of any word is interconnected with the meaning of other words, while semantic atomism holds the view that the meaning of a word is detached from the meaning of other words; its meaning stands on its own, as it were.

Minimal and maximal literalists, despite their differences, agree on the centrality of apparent meaning. For them, the apparent meaning of any concept is fixed only on the “designation” (*waḍ‘*) and for them this is the key to understanding the meaning of the text. For example, the concept of “mother” is designated for the one who carries the fetus to term. Its meaning then is irrelevant to other concepts and to the context of use, assertion, and comprehension. As mentioned above, this position is called semantic atomism. Literalists are semantic atomists.

But, for semantic holists, what determines the meaning of a concept is not only the matter of designation, nor it is the key to understanding the meaning of the text, but it is mainly determined by a web of interconnected meanings as well as by the context in which it is produced and also the context in which it is asserted and comprehended.

More importantly, this context may differ (even for a single person) from time to time and place to place, and since the meaning of a concept is not detached from other concepts, a change in a concept might bring changes to other concepts. For example, due to the technologically advanced context in which we live, the concept of “mother” has been expanded to literally denote not only the one who carries the fetus to term but also the one who produces ova. If, for literalists in religious context, the meaning of the word when the sacred text is revealed is the key, then they run into difficulty as the meanings of words constantly, fundamentally, and unavoidably evolve over the time.

Semantic holism has a revolutionary effect on religious hermeneutics. Taking linguistic meaning to be indeterminate and open-textured requires that no priority is given to the apparent (designated for the first time) meaning of a linguistic expression. As is the case with concrete concepts such as “mother,” it is significantly more so with abstract concepts such as “God,” “God-mindfulness” (*taqwā*), “salvation,” and so on.

Semantic holism is anti-literalistic altogether. For semantic holists meaning is not discovered, it is recreated in each and every different context, although there might turn out to be a family resemblance between different meanings. Let us call this the “impossibility of literal interpretation” critique. This critique could significantly challenge literalism of any sort, maximal or minimal. Unless literalists provide a plausible defense of literalism against the challenge, even minimal literalism cannot be taken as face value.

### Contextualist Approaches

#### Analysis

In the absence of explicit legal permission or prohibition in the scriptures, the contextualists, unlike textualists, neither limit their judgement nor extend the textual boundaries but make context-oriented claims in their deliberation on biomedical ethical issues. The contextualists consider the jurisprudential, theological, philosophical, and social “contexts” of the scriptural text, together with extra-scriptural legal and moral sources within Muslim intellectual history (Rasekh and Khodaparast 2013). Understood in this way, if someone claims that context should play a vital role in the hermeneutical understanding of the scriptures, a legitimate question appears: how and from where should one derive these contexts? There are, broadly speaking, two ways in which this question is settled among Muslims. Some *derive* their interpretations and contexts from *within* the jurisprudential corpus that has been handed down throughout the centuries and believe that context-oriented Muslim legal theory is enough to be used for interpreting the scriptures. However, there are others who are willing to go beyond the tradition of Muslim legal theory, or at least seek to amend it, by giving a substantive place to philosophical-moral reasoning. The former stance is what we label as a contextualist approach to biomedical ethics. The latter stance is that of the para-textualists, which will be examined shortly. Even though the para-textualists are also contextualists of a kind, we reserve the term contextualist here for approaches that tend to adopt and develop a methodology with which we can derive biomedical ethical solutions *only* from within the Muslim legal tradition (Shabana 2014; Bagheri 2011). In this paper, we use the term Sharīʿa and Sharīʿa law interchangeably to designate the corpus of this collected Muslim legal tradition. By Sharīʿa or Sharīʿa law we mean moral/legal deliberations coming from within the enterprise of *fiqh* and *uṣūl al-fiqh*. Sharīʿa as an abstract concept also sometimes refers to what is willed by God, but our reference here is to its approximations or representation within Muslim jurisprudence.

Contemporary scholars who seek to derive biomedical ethical solutions *only* from within the Muslim legal tradition tend to believe that Sharīʿa has intrinsic value, and that biomedical ethical solutions should be derived from within it. Their attempt is to revive, reinterpret, and refine Sharīʿa as a whole, incrementally with reference to Shīʿī or Sunni legal theories, to infer new biomedical solutions in a way that is both Sharīʿa-compliant and compatible (as far as possible) with modern life (Bagheri and Afshar 2011). Such revival, reinterpretation, and refinement of Sharīʿa law has led the traditional Muslim scholars to declare new legal opinions (*fatwā*s) on biomedical issues—legal opinions that cater to the context in which they live. For example, there are scholars who, in their argument, apply the long-established legal maxim of “no harm and no harassment” (*lā ḍarar wa lā ḍirār*) to issue a legal opinion in favour of euthanasia under specific circumstances (Sachedina 2006, 2009; Brockopp 2003; also see Shubayrī-Zanjanī’s *fatwā*, as a contemporary Twelver Shīʿī authority, on euthanasia based on the *lā ḍarar* principle in Shafaqna 2022). There are others who develop new pragmatic definitions, such as that of brain death by reference to the traditionally established juristic category of “unstable life” (*al-ḥayāt ghayr al-mustaqirra*), to find a solution for circumstances where the removal of life support can be permitted (Padela, Arozullah, and Moosa 2013; Moosa 1993). Interestingly, the Shīʿī legal scholars (*fuqahāʿ*) in Iran used this line of argument and passed the Organ Transplantation and Brain Death Act approved by Iran’s parliament in 2000. The argument makes a distinction between the stable and the unstable states of life. The stable state of life is the conscious and normal state, while the unstable state of life is a life closer to death and less dignified. This distinction is then used to explain why organ transplantation from brain-dead donors is permissible. If a transplant of vital organs, such as the heart, is necessary for saving a life, it is permissible to end a brain-dead individual’s life and use his/her organs for transplantation (Aramesh et al. 2018; Mahdavi-Mazdeh 2012).

Even though all such jurists and scholars rely heavily on the scripture and use context-oriented legal theory to reach their conclusions, two further divisions can be made within contextualist approaches. Following contemporary scholarship on Muslim biomedical ethics, we can make a methodological distinction between “narrow” and “broad” approaches in the contextualist camp. Within the narrow approach, the contextualists mostly focus on the so-called detailed rulings of positive law (*aḥkām tafṣīliyya*) in the various branches of Muslim jurisprudence (*furūʿ al-fiqh*) to deliberate on the permissibility and impermissibility of biomedical ethical issues (Ghaly 2013; Hamdy 2012; Clarke, Eich, and Schreiber 2015; al-Khaṭīb 2020; for a critique of the “fatwā-centred” approach, see Sing 2008). Within a broader approach, the contextualists focus on the higher objectives of Sharīʿa (*Maqāṣid al-Sharīʿa*), which are typically taken to be the preservation of 1) religion, 2) life, 3) intellect, 4) progeny, and 5) wealth. In this broader approach, searching for a new legal opinion is not the primary aim. What matters most is refining and reviving the objectives of Sharīʿa to make it relevant to addressing new biomedical issues (for a discussion of *Maqāṣid al-Sharī‘a*, see Auda 2008, 2016; Opwis 2010). For example, some contextualists taking the broader approach have even determined that the four principles of bioethics proposed by Beauchamp and Childress (2001), that is, respect for autonomy, beneficence, non-maleficence, and justice, are compatible with the higher objectives of Sharīʿa (Atighetchi 2007; Aksoy and Tenik 2002; Aramesh 2008; al-Bar and Chamsi-Pasha 2015).

Other trends in the wider contextualist camp render the *adab* literary tradition in Muslim history within the scope of higher objectives of Sharīʿa. In this approach, Muslim bioethicists seek to revive an *adab*-oriented Muslim bioethics which generally refers to issues such as the acquisition of virtues which the physicians should practice in their field (Sartell and Padela 2015; Nanji 1988; Cuceu at al. 2020; Brockopp and Eich 2008; al-Ghazālī 1963). However, such contextualists tend to assume that the learnings gleaned from *adab* literature are not sufficient for deliberating on biomedical issues, as ethics should be understood only with reference to Sharīʿa and the jurisprudential framework within Islam (Daar and Khitamy 2001; Daar, Bakdash, and Khitamy 2008). According to this hybrid approach, *adab* literature works as a means of reaching those ends which are already outlined within the higher objectives of Sharīʿa (Padela 2007). The goal here is to subsume Islamic ethics under the Sharīʿa-based duties and obligations (Reinhart 1983).

This is an important point to note in the contemporary wider contextualist trend in Muslim biomedical ethics. The ethical considerations which lie outside the domain of jurisprudence are not treated as independently valid for treating biomedical ethical issues, since the whole ethical system should be defined with reference to Sharīʿa alone. For example, if euthanasia is impermissible with reference to Sharīʿa, it must also be so in light of the moral arguments from within the Muslim ethical traditions (Shomali 2008). If moral arguments from the Muslim ethical tradition considered euthanasia to be permissible, whereas Sharīʿa considered it impermissible, the contextualists would not consider that permissibility to be legitimate. Thus, contextualists believe that only Sharīʿa-based religious norms are essential for the legitimacy of Muslim biomedical ethics. Any other norms, based on cultural or even moral precedents, are to be secured by the standards of Sharīʿa (Shabana 2013).

#### Critique

Muslim contextualists tend to believe that moral perceptions and considerations make sense *only* in light of, and in terms of, Sharīʿa law principles. Moral considerations should not be understood independently of Sharīʿa law. For example, contextualists could argue that it is prima facie permissible to harm an aggressor in defending yourself, only because Sharīʿa law commands us to do so and not because we have an independent moral reason that may prescribe the same. Let us call this view Sharīʿa command theory, according to which moral requirements are only determined by Sharīʿa commandments. These are the *only* means of discerning what is morally permissible or impermissible. It is not the case that Sharīʿa commands us to do certain kinds of actions because they are morally required. The theory states the opposite. That is, certain kinds of actions are morally required because Sharīʿa commands them.

Muslim contextualists do not only accept that whatever is morally required (or morally good) is so because Sharīʿa commands it, but they also believe that Sharīʿa law commands us to do certain kinds of acts because they are morally required or good. This is *logically* inconsistent with acceptance of Sharīʿa command theory. If Sharīʿa commands us to do certain kinds of acts because they are morally good, then Sharīʿa should first recognize what is morally good prior to commanding it. This, however, entails that Sharīʿa does not make it morally good—it was already morally good before Sharīʿa commanded it. To say this is to accept that there are criteria of moral goodness independent of Sharīʿa’s commands. Yet, Sharīʿa command theory rejects this view, since moral goodness is not considered independent of Sharīʿa commandments.

The burden of resolving this logical inconsistency falls on the shoulders of contextualists. Whether they believe that the commandments of Sharīʿa are morally good, or that the Sharīʿa is intrinsically morally good, they must explain why there is some criterion of goodness independent of Sharīʿa.

### Para-Textualist Approaches

#### Analysis

Like contextualists, para-textualists take “context” seriously to find solutions for modern biomedical issues. However, unlike contextualists, para-textualists are not necessarily looking to make a *compromise* between the scriptural sources and moral deliberations on biomedical ethical issues. Rather, they intend to *reconstruct* an independent rational framework for biomedical issues on the condition that it does not neglect the core ethical vision of the scriptural sources. For para-textualists, however, seeking *compliance* with the scriptural sources is not unconditionally, prima facie, at stake. Instead, compliance with the scriptures is ultimately at stake only if the scripture is seen to make rational claims. Para-textualists believe that human beings can evaluate scriptural claims using independent reason. To this end, in their methodology, para-textualists seek to recognize reason as a substantive source for ethical deliberations besides scripture. However, this does not imply that these solutions are necessarily contrary to Sharīʿa.

Para-textualism is an emerging approach within Muslim biomedical ethics, and as such has not yet developed to the extent that textualists and contextualist tendencies have developed within Muslim biomedical literature. This is not to say that the para-textualist approach in general is in its infancy. There is a long tradition within both classical and contemporary Muslim philosophy (*falsafa*) and Muʿtazilī-inspired theology (*kalām*) that gives a substantive role to reason vis-à-vis scripture in moral deliberations (Ahmed 2015; Panjwani and Revell 2018; Akhtar 2007; note that these scholars’ approaches are not necessarily about Muslim biomedical ethics). For example, para-textualism can be found in the works of Aristotelian-inspired Muslim philosophers such as Ibn Miskawayh (d. 1030) and Naṣīr al-Dīn al-Ṭūsī (d. 1274), who focused extensively on the concept of true happiness or eudaimonia (*saʿāda*) (Naraqi 1987; Al-Ghazālī 2001; Al-Ghazālī was also among those philosophers who invested a lot in the concept of *saʿāda*, though he was not Aristotelian). The concept of *saʿāda* for these philosophers works as a *normative* concept according to which one can deliberate about what constitutes the good life (*al-ḥayāt al-ṭayyiba*) (Nanji 1991). Although the concept of *saʿāda* is philosophically independent of Sharīʿa, this does not imply that the ethics of *saʿāda* are necessarily against Sharīʿa. For example, the case of *al-ḥayāt al-ṭayyiba* can be approbated in the Qur’an (See Q16:97). If we consider Sharīʿa in its wider sense then it is not the case that the concept of *saʿāda* is philosophically independent of Sharīʿa, rather it lies within its confines even if outside the legal domain of Sharīʿa.

Thus, within para-textualist approaches, Muslim bioethicists take independent moral reasoning seriously. Unlike contextualist approaches, in which moral reasoning is derived from and should *necessarily* be read in light of Sharīʿa, in para-textualist approaches moral reasoning and Sharīʿa both have weightage when they come into conflict. The para-textualist tendency is towards reconstructing Sharīʿa rather than refining it by having recourse to sources other than the scriptural sources for such reconstruction. For example, para-textualists will not use virtue ethics–based *adab* literature as a means of refining the higher objectives of Sharīʿa, as in the case of the contextualists. Rather, para-textualists will tend to use independent philosophical reasoning to reconstruct Sharīʿa when deliberating on biomedical ethical issues.

In para-textualist approaches, philosophical reasoning about biomedical issues and solutions has an intrinsic value and need not be derived from Sharīʿa. Through reconstructing the edifice of Sharīʿa in light of independent moral considerations, para-textualists believe that Sharīʿa and moral considerations must reflectively reach an equilibrium (John Rawls 1971; Scanlon 2002). For example, one might argue that if we have a justified moral reason to believe that abortion is permissible, we could consider abortion as an option to perform as long as there is no absolute rejection (*ḥurmat al-mukhālafa al-qaṭʿiyya*) of performing it in Sharīʿa or if there are peer disagreements about it among scholars (Alishahi Tabriz, Dabbagh, and Koenig 2016; The Brussels Collaboration on Bodily Integrity 2019, Dabbagh 2022). In fact, for para-textualists, peer disagreement can be considered an epistemic virtue when arguing about a particular biomedical ethical issue while accepting the authority of the Qur’an and Sunna (Dabbagh 2017). This is because peer disagreement could give para-textualists good reason to argue about a particular case independently of Sharīʿa, when there is no uniform answer or consensus derived from Sharīʿa. In addition to moral philosophy, para-textualists also rely on contemporary literature in the fields of philosophy of mind and biology to discuss, for example, how the concept of death should be defined normatively for Muslims (Rasekh and Ayati 2007). Muslim bioethicists have also invoked the traditionally accepted discretionary sphere in Muslim legal principles (*minṭaqat al-farāgh*) to argue about the validity of their approaches (Aramesh 2020). Such discretionary spheres have been traditionally accepted within the Muslim juristic enterprise in areas where Sharīʿa is found to be silent or where it is not clear what the judgement (*ḥukm*) should be. In such instances, a valid case can be made for independent moral reasoning. For example, policymakers in the health sector in Iran have used the theory of *minṭaqat al-farāgh* to create ethical guidelines and legal regulations for certain practices such as organ transplantation and abortion. Although these ethical guidelines are Sharīʿa independent, they have been endorsed by traditional Muslim legal scholars (Aramesh 2007; Zahedi and Larijani 2008).

Let us suppose that a para-textualist wants to argue for a theory about organ distribution based on the ethics of organ donation from a Muslim perspective. A para-textualist starts to argue for an independent theory of fairness, for instance, based on drawing lots to assign indivisible goods (e.g., organs) to different people who have equally strong competing moral claims. In cases where people’s claims on an indivisible good are equally strong, fairness requires that the good ought to be distributed by lottery. In the next step, the para-textualist argues then that this line of argument is not incompatible with what is referred to in *al-Qawāʿid al-Fiqhiyya* (Islamic Legal Maxims) as the Principle of Drawing Lots (*Qāʿidat al-Qurʿa*). According to this principle, in both Shīʿī and Sunni schools of Islamic jurisprudence, if it is hard to make a judgement, it is reasonable to appeal to lottery, chance, or luck for making a judgement. Scholars who advocate such a principle often refer to Qur’anic narratives in which people draw lots in the process of decision-making. For example, in the process of adopting Maryam, they draw lots (Q3:44) or in the case of Yunis, the men cast lots and he was among the losers to be thrown out of the ship (Q37:141).^5^

Before concluding this section, we must make an important point: the distinction presented here between contextualist and para-textualist approaches should not be considered a hard distinction. This is also the case with the distinctions between textualist and contextualist approaches. What we have instead is a *spectrum* of different approaches. Hence, there might be cases that sit on the boundaries of these approaches and some borderline scholars might not fit entirely into these categories. For example, theologically speaking, almost all Para-textualists tend to embrace a Muʿtazilī-inspired theology, while it is not the case that all contextualists *only* espouse an Ashʿarī-inspired theology. For example, one might be a contextualist but not a fatalist. A contextualist might argue that it is up to God to decide what is good and bad but it is up to humans to obey or disobey him (Muʿtazilī and Ashʿarī approaches in ethics can also be applicable in *uṣūl al-fiqh*. For more on this, see Johnston 2004). There are scholars whose methodology oscillates between the para-textualist approach and the wider contextualist approach identified above, who tend to embrace a Muʿtazilī-inspired theology. There are also scholars, such as Fazlur-Rahman (d. 1988), Abdullahi Ahmed An-Na’im, and Ebrahim Moosa, some of whose works fit into contextualist approaches while their other works fit into para-textualist methodology (Rahman 1998; An-Na’im 1990, 2010; Moosa 2007; Panjwani 2012).

As an example, in his *Health and Medicine in the Islamic Tradition*, Fazlur-Rahman argues at length against the “anti-intellectualist” Ashʿarī trend among Muslim scholars. However, for biomedical issues, like many contextualists, his point of departure is Qur’anic moral guidance and the Sunna. He, for example, argues that there are certain developments in Muslim biomedical ethics which can be considered as deviations from Qur’anic holistic norms towards the practice of medicine (Sonn 1996). He even refers to five fundamental “rights” in Islamic law, i.e., “preservation of life, religion, property, personal honor, and sound mind” that are guaranteed to all citizens (Rahman 1998, 70). What Fazlur-Rahman is referring to here is the notion of the higher objectives of Sharīʿa which occupies the approach of contextualists discussed above. Although Fazlur-Rahman, in his biomedical ethics, is methodologically closer to the contextualist approach for deriving solutions to these problems from within the scriptures, his reformist attitude self-professedly rejects Ashʿarī-inspired theology.

#### Critique

Perhaps one of the most frequently repeated criticisms against para-textualists is that this rationally independent approach is not *Islamic* enough. This is because the point of departure for solving biomedical issues within this approach is not scriptural sources but rather independent moral reasoning, whether found in Western philosophy or in Muslim jurisprudence or elsewhere. Yet, looking at para-textualists’ writings, such criticism is not valid. It is correct to say that para-textualists do not have scriptural sources as their point of departure. However, they approbate the scriptural text and return to it to show that their rational claims are in tune with the scripture. In other words, para-textualists try to make sure that their arguments do not categorically rebut existing claims in the scriptural sources. Hence, their approach is not un-Islamic, at least not in the sense of being detached from the ethical core of the scripture.

However, even if the para-textualists’ approach is not un-Islamic, its methodology does not seem fully compatible with what is perceived as an “orthodox” approach towards the scriptures. In giving a substantive role to reason, most para-textualists depart from the common attitude in giving primacy to Sharīʿa. Even though there are several Qur’anic verses in support of reasoning and rationality (e.g., Q6:50), one might argue that the spirit of the Qur’an has mostly been understood to date in line with the Ashʿarī mode of thinking. For if God is omniscient according to the Ashʿarīs, then God knows what is best and just—who are we to judge what is just or not? (Al-Juywanī 2000). Following an Ashʿarī mode of thinking implies that using our independent moral reasoning to argue for what is right or wrong is futile.

Moreover, one might argue against para-textualists that separating ethics from Sharīʿa is not plausible, because, at least historically speaking, ethics and Sharīʿa have not been separated from one another. They have evolved in tandem, with Sharīʿa having been seen as consisting of what Muslims need for a moral life. Thus, contextualists would argue that ethics should be understood in light of Sharīʿa laws alone and there is no need to think of an independent moral framework other than existing moral principles within Sharīʿa. However, this should not be a problem for para-textualists because, like many Ashʿarīs, they also believe that a *shāriʿ* (legislator) should take morality into account before making a normative religious judgement (*ḥukm*). Hence, Sharīʿa and morality, at least in principle, go hand in hand. This is the minimum that both Ashʿarīs and Muʿtazilīs agree on. However, para-textualists who tend to embrace a Muʿtazilī approach in their theology are not like Ashʿarīs because they try to open the possibility of morally criticizing religious judgements by separating ethics from Sharīʿa. Even though ethics and Sharīʿa have not been separated in the course of history, this does not necessarily mean that they cannot be separated theoretically. Separating ethics from Sharīʿa enables para-textualists to interpret scripture outside the confines of literalism and the inconsistency of Sharīʿa command theory.

## Conclusion

There is widespread recognition among Muslim scholars dealing with biomedical ethical issues that context plays an essential role in forming ethical principles and judgements. The context-sensitive approaches in Muslim biomedical ethics respond to the requirements of modern biomedical issues by recognizing the contexts in which scriptural text has been formed and developed through the course of Muslim intellectual history. This paves the way for bringing in different context-sensitive interpretations of the sacred texts through different reasoning tools and methods, whether they are rooted in the *uṣūl al-fiqh* tradition for the contextualists, or in moral philosophy for the para-textualists. For the textualists, reasoning outside of the textual boundaries is not acceptable. While contextualists tend to believe that contextual considerations make sense only in light of Sharīʿa law and should not be understood independently of Sharīʿa law, para-textualists believe that moral perceptions and contextual considerations are valid irrespective of Sharīʿa law, insofar as they do not neglect the moral vision of the scriptures. The common ground between the majority of the textualists and the contextualists lies in giving primacy to the Sharīʿa law. Moral requirements for both the textualists and the contextualists are only determined by Sharīʿa commandments, and Sharīʿa commandments are the *only* basis on which to decide what is morally permissible or impermissible in biomedical ethical issues. This is an Ashʿarī-inspired approach to biomedical ethics with respect to human moral reasoning (Sachedina 2005; Aramesh 2020; Reinhart 2004; Moosa 2004; Moosapour et al. 2018).

Para-textualists, on the other hand, do not deny the relevance of Sharīʿa, but treat the reasoning embedded in Sharīʿa as being on a par with moral reasoning in general. Thus, if there are contending strands of moral reasoning on a particular biomedical ethical issue, Sharīʿa-based reasoning will need to compete with other moral reasoning on the issue. If the *aḥkām* (religious judgements) are deemed to be reasonably sound, then for para-textualists there are no grounds for not accepting them. Although using and referring to Sharīʿa might work in many cases, it is not the case that Sharīʿa is enough in *every* case to judge on moral issues. For instance, morally speaking, it is not enough to refer to Sharīʿa when someone is choosing or refusing euthansia or abortion. For para-textualists what matters most is *how* Sharīʿa morally reasons about the permissibility or impermissibility of an action. If it is morally justified to euthanize or abort, we are rationally (and morally) bound to accept it, and if it is not morally justified, we will then either have to leave our judgement about choosing or refusing euthanasia or abortion or find another context-sensitive interpretation to rationalize the relevant commandment derived from Sharīʿa. Thus, the departure point for the para-textualist approach is moral reasoning, whether it is found in moral philosophy, Muslim jurisprudence, or elsewhere (Soroush 2009; Shahrur 1990, 2009; Hallaq 1997; An-Na’im 2008). Para-textualist methodology tries to remain open to the possibility of morally criticizing religious judgements (*aḥkām*), while remaining true to the moral vision of the scriptures. This is a Muʿtazilī-inspired approach to biomedical ethics (Hourani 1976; Vasalou 2008; Sheikh 2019; Farahat 2019; Reinhart 1995; Al-Bar and Chamsi-Pasha 2015; Hallaq 2014).

## Footnotes

1 *Naṣṣ* and *ẓāhir* are categorized together under the exegetical category of *muḥkam* (clear), whereas *muʾawwal* and *mujmal* are categorized under *mutashābih* (ambiguous). *Muḥkam* and *mutashābih* are the terms mentioned in the Qur’an (Q3:7). For more information, see al-Ṣadr 2003, 173–194.

2 Akhbāriyya was a jurisprudential movement within Twelver Shīʿī tradition, initiated by Muhammad Amin al-Astarabadi (d. 1036). For a short review of Akhbāriyya, see Gleave 2018, 207–230; al-Ṣadr 2003. Uṣūlīs (lit. principalists) form the dominant branch of Twelver Shī‘ī legal scholars who apply the rationalist principles to the legal hermeneutics of revelation (Newman and Jansen 2012). For interpreting the Shīʿī uṣūli approach as a kind of literalistic approach, see Habib 2010, 71–72.

3 For a comprehensive encyclopaedia detailing with Sunni legal ruling in biomedical cases accompanied by text proofs from the Qur’an and/or prophetic hadith, see Kanʿān 2000. For an informative summary of the diversity of Sunni legal rulings on abortion, in most of which textualism can be found, see the Centre for Contemporary Islamic Jurisprudence 2014, 12–35.

4 Muḥammad Qāʾinī (b. 1965) is a prominent contemporary Twelver Shīʿī jurist from Iran.

5 Our argument in this paragraph is inspired by Hossein Dabbagh’s talk, “Ethics of organ transplantation: ‘Qaʿidat al-Qurʿa’ as the Basis for Fair Practice,” delivered at Al-Mahdi Institute’s “Islamic Perspectives on Organ Donation after Death” Research Workshop, Birmingham, United Kingdom, 2019.
